# Titanium Surface Properties Influence the Biological Activity and FasL Expression of Craniofacial Stromal Cells

**DOI:** 10.1155/2019/4670560

**Published:** 2019-01-13

**Authors:** Enrico Conserva, Alessandra Pisciotta, Francesco Borghi, Milena Nasi, Simone Pecorini, Laura Bertoni, Anto de Pol, Ugo Consolo, Gianluca Carnevale

**Affiliations:** ^1^Department of Surgery, Medicine, Dentistry and Morphological Sciences with Interest in Transplant, Oncology and Regenerative Medicine, University of Modena and Reggio Emilia, Modena, Italy; ^2^Department of Biomedical, Metabolic and Neural Sciences, University of Modena and Reggio Emilia, Modena, Italy

## Abstract

Mesenchymal stromal cells (MSCs) can be easily isolated form craniofacial bones during routine dentistry procedures. Due to their embryological origin from neural crest, they represent a suitable cell population to study cell-biomaterial interaction in the craniofacial field, including osteoinductive/osteointegrative processes. The biological and immunomodulatory properties of MSCs may be influenced by chemistry and topography of implant surfaces. We investigated if and how three different titanium surfaces, machined (MCH), sandblasted with resorbable blasting medium (RBM), and Ca^++^-nanostructured (NCA), may affect biological activity, osseointegration, and immunomodulatory properties of craniofacial MSCs. Cell proliferation, morphology, osteogenic markers, and FasL were evaluated on MSCs isolated from the mandibular bone after seeding on these three different surfaces. No statistically significant differences in cell proliferation were observed whereas different morphologies and growth patterns were detected for each type of surface. No difference in the expression of osteogenic markers was revealed. Interestingly, FasL expression, involved in the immunomodulatory activity of stem cells, was influenced by surface properties. Particularly, immunofluorescence analysis indicated that FasL expression increased on MCH surface compared to the others confirming the suggested role of FasL in promoting osteogenic differentiation. Titanium surface treatments and topography might reflect different biological behaviours of craniofacial MSCs and influence their osseointegration/immunomodulation properties.

## 1. Introduction

The interactions between cells and implant surfaces play an essential role in the field of dental surgery. Osseointegration process consists in the ability of bone marrow mesenchymal stem cells to adhere to the implant and becoming mature osteoblasts [1]. It is well known that this process is influenced by different events, such as the micro/nanotopography and the chemical composition of implant surfaces [2–4]. As a matter of fact, the roughness of the implant surface and its chemistry, topography, and energy/wettability were demonstrated to impact cell biological properties [5]. Different cell sources have been studied *in vitro* to evaluate how surface properties might affect osseointegration [6]. In recent years, interest has grown significantly in stem cell research due to stem cell ability to regenerate lost and injured tissue, especially in the craniofacial field, where the rehabilitation of this complex anatomical district represents a challenging situation for surgeons and dentists [7, 8]. Mesenchymal stromal/stem cells (MSCs) are commonly identified by a distinct surface phenotype as well as by their potential to differentiate into specific lineages *in vitro* [9]. Compared to bone marrow MSCs (BM-MSCs) isolated from axial and appendicular bones, craniofacial MSCs derived from the neural crest [10] exhibit distinct properties, either when cultured *in vitro* [11, 12] or when heterotopically transplanted *in vivo* [13]. In fact, faster cell proliferation, delayed senescence, higher expression levels of alkaline phosphatase, and calcium accumulation, combined with a lower tendency towards adipogenic and chondrogenic differentiation, were reported [12]. To this regard, it has been suggested that the selection of the most appropriate stem cell source is crucial for a successful cell-based therapy for skeletal healing of different anatomical regions [14]. It has been also widely demonstrated that the expression of Fas and FasL, important mediators of apoptosis, gives to craniofacial stem cells the capability to modulate the immune/inflammatory response [15–17] and the osteogenic commitment [18]. Fas ligand is a transmembrane protein that belongs to the TNF family and seems to play a role in the maintenance of physiological bone mass by inducing osteoclast apoptosis [19]. FasL expression in craniofacial stem cell has been investigated by different research groups [20]; however, it is still unknown whether its expression can be influenced by the different titanium surface properties, such as surface roughness and chemistry. The aim of this study was to investigate the biological activity and FasL expression in craniofacial MSCs cultured on different titanium surfaces.

## 2. Materials and Methods

### 2.1. hBM-MSC Collection and Immunophenotypical Characterization

**Figure 1:**
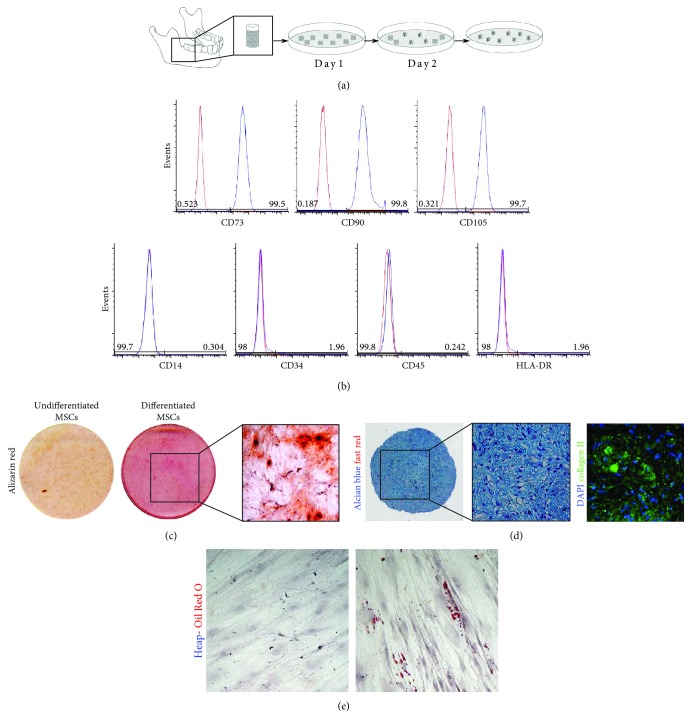
Isolation, characterization, and differentiation of MSCs. (a) Schematic representation of MSCs isolated from the mandibular bone and expanded *in vitro*. (b) Immunophenotypical characterization performed through FACS analysis on MSCs against mesenchymal surface antigens. (c) Alizarin red staining carried out on undifferentiated and osteogenic differentiated MSCs. (d) Alcian blue and fast red staining on MSCs induced towards chondrogenic differentiation. On the right, immunofluorescence analysis shows positive staining for collagen II in chondrogenic differentiated MSCs. (e) The presence of lipid-rich vacuoles was evaluated through Oil Red O staining in undifferentiated and differentiated MSCs.

The study was carried out in accordance with the recommendations of the ethics committee of the province of Modena (Italy). Human MSCs were isolated from the mandibular bone, as previously described by Carnevale et al. [21], from patients (*n* = 3; 35 to 40 years old) undergoing routine implant surgery in the mandible, after obtaining their written informed consent, in compliance with the Declaration of Helsinki (Figure 1(a)). MSCs at passage 1 (P1) were stained for cell surface markers and analyzed by flow cytometry, as reported in Supplementary Materials (available here).

### 2.2. Multilineage Differentiation Assays

MSCs (P1) were used for the induction towards osteogenic, adipogenic, and chondrogenic commitment *in vitro*, as described in Supplementary Materials.

### 2.3. Titanium Surface Characterization

A total of 105 titanium disks, directly provided by the manufacturer (MEGAGEN Co. Ltd., South Korea), measuring 8 and 13 mm in diameter and 3 mm in thickness were used in this study. Three different titanium surfaces were investigated: machined (MCH), sandblasted with resorbable blasting medium (RBM), and sandblasted with a Ca^++^-incorporated nanolayer (NCA). The treatment processes are hold by the manufacturer. Surface morphology was qualitatively evaluated using a scanning electron microscope (EVO MA 10, Carl Zeiss, Oberkochen, DE) working at 25 keV. At the same time, surface chemistry was analyzed by using an energy dispersive X-ray spectrometer (EDX). Furthermore, surface roughness was assessed using an atomic force microscope (Nanoscope IIIa, Veeco, Plainview, USA); then, roughness average (Ra) and roughness peak-to-valley (Rpv) parameters were obtained. Ra measures the average surface roughness considering the peaks and the valley means. The Rpv describes the maximum observed range in a sample area, and it is given by the distance between the highest peak and the lowest valley on a measured surface.

### 2.4. Cell Morphology and Proliferation

Undifferentiated mandibular MSCs (P1) were seeded at a density of 2.5 × 10^3^ cells/cm^2^ on titanium disks in a 12-multiwell unit. For each experimental time (1, 2, 4, and 7 days), three disks of MCH, RBM, and NCA were used. Cells were cultured in standard conditions and maintained in an expansion medium (*α*-MEM, 20% FBS, 1% L-glutamine, 1% penicillin and streptomycin, all from Sigma-Aldrich, Saint Louis, MO, USA). At the end of each experimental time, the cells were fixed in ice-cold paraformaldehyde 4% for 15 minutes without dissociating them from the titanium disks. The cells were subsequently permeabilized with 0.1% Triton X-100 in PBS for 5 minutes, stained with TRITC-conjugated anti-phalloidin antibody (Life Technologies, Carlsbad, USA), and rinsed with PBS. Nuclei were stained with 1 *μ*g/mL 4′,6-diamidino-2-phenylindole (DAPI) in PBS. DABCO was used as an antifading mounting medium. Cell proliferation and morphology were assessed through a Nikon A1 confocal laser scanning microscope. Cell proliferation was evaluated by counting the DAPI-stained nuclei on 10 randomly selected fields measuring 2.85 × 10^5^ *μ*m^2^ for each disk by a blind operator.

### 2.5. Osteogenic Induction

In order to evaluate the ability of the surfaces to influence osteogenic differentiation, cells were seeded at approximately 2.5 × 10^3^ cells/cm^2^ on the MCH, RBM, and NCA surfaces. After one week of culture, the standard culture medium was replaced with the osteogenic medium as described above. After 3 weeks of induction, the expression of typical differentiation markers, such as RUNX2, osterix (OSX), osteocalcin (OCN), and secreted phosphoprotein 1 (SPP1), was evaluated by immunofluorescence and real-time polymerase chain reaction (real-time PCR) analyses.

### 2.6. Immunofluorescence and Confocal Microscopy

Cells were fixed in 4% ice-cold paraformaldehyde in PBS for 20 minutes, permeabilized with 0.1% Triton X-100 in PBS for 5 minutes, and then processed as previously described by Carnevale et al. [22]. The following primary Abs diluted 1 : 100 were used: mouse anti-OCN and rabbit anti-Runx-2 (Abcam, Cambridge, UK), rabbit anti-osterix (OSX; GeneTex, San Antonio, TX, USA), rabbit anti-collagen II (Abcam), and rabbit anti-FasL (Cell Signaling Technology, Leiden, NL). Secondary Abs (goat anti-rabbit Alexa 488, goat anti-mouse Alexa 488, and goat anti-mouse Alexa 546; Life Technologies) were diluted 1 : 200. Nuclei were stained with 1 *μ*g/mL DAPI in PBS. Negative controls consisted of samples not incubated with the primary Abs. The multilabeling immunofluorescence experiments were carried out avoiding cross-reactions between primary and secondary Abs. Confocal imaging was performed by using a Nikon A1 confocal laser scanning microscope. The serial sections were processed with ImageJ software to obtain three-dimensional projections, and image rendering was performed by Adobe Photoshop Software. Staining intensity of FasL expression in MSCs seeded on different titanium surfaces was evaluated by pseudocolor analysis: blue to white arrays the colors in a spectrum with blue assigned to a lower value than white.

### 2.7. Expression of SPP1 and RUNX2 mRNA

Total RNA was extracted from cells cultured on titanium disks after 3 weeks of osteogenic induction using Quick-RNA MiniPrep (Zymo Research, Irvine, CA, USA) following manufacturer instruction. RNA concentration has been assessed measuring absorbance at 260 nm by NanoDrop ND-1000 (Thermo Scientific, Waltham, MA, USA). Samples were reverse transcribed with iScript cDNA synthesis kit (Bio-Rad, CA, USA). The mRNA expression was measured by real-time PCR using a mix that consisted of 2x SsoAdvanced Universal SYBR Green Supermix (Bio-Rad), 1 *μ*L of prevalidated sets of PrimePCR™ SYBR® Green Assay (Bio-Rad), and 1 *μ*L of cDNA and sterile, distilled water to a 20 *μ*L final volume. Each reaction was performed in triplicate in a CFX96 Touch Real-Time PCR (Bio-Rad). The following primers have been used to quantify gene expression: SPP1 and RUNX2 (Bio-Rad; ID: qHsaCID0012060 and qHsaCED0044067). GADPH and RPS18 (Bio-Rad; qHsaCED0038674 and qHsaCED0037454) have been evaluated as housekeeping genes. Using the *∆*Ct method, results are reported as expression relative to the most stable housekeeping gene GADPH.

### 2.8. Statistical Analysis

GraphPad Prism (GraphPad Prism Software Inc., v5) was used to perform statistical analysis. Each experimental evaluation was performed in triplicate. Significance was assessed by analysis of variance (ANOVA). Values of *P* < 0.05 were considered as statistically significant.

## 3. Results

### 3.1. Immunophenotypical Characterization

After reaching the confluence, the expression of MSC surface antigens was evaluated on adherent cells by flow cytometric analysis. As reported in Figure 1(b), at passage 1, almost all MSCs isolated from mandibular fragments were positive for CD73, CD90, and CD105 mesenchymal cell surface antigens. Conversely, cells did not express the hematopoietic and endothelial markers CD16, CD34, and CD45 and the class II major histocompatibility complex (MHC) cell surface receptor HLA-DR (Appendix Table 1). These data confirmed the mesenchymal phenotype of the cells used for the study.

### 3.2. Multilineage Differentiation Potential

The ability of isolated cells to differentiate *in vitro* along osteogenic, adipogenic, and chondrogenic lineages was assessed after 3 weeks of culture in the appropriate media.

#### 3.2.1. Osteogenic Differentiation

After 3 weeks of induction in the osteogenic culture medium, the *in vitro* deposition of mineralized extracellular matrix was evaluated by Alizarin red staining. Spots of mineralized extracellular matrix were detected in differentiated MSCs. Undifferentiated cells were negative (Figure 1(c)).

#### 3.2.2. Chondrogenic Differentiation

Chondrogenic differentiation was performed in pellet culture system. After three weeks of induction, pellet sections were labeled with Alcian blue/fast red stain. As reported in Figure 1(d), a detectable extracellular deposition was observed. Moreover, immunofluorescence analysis revealed the expression of collagen II by the committed cells.

#### 3.2.3. Adipogenic Differentiation

The evident morphological shifts in MSCs were characterized by the accumulation in the cytoplasm of lipid drops clearly stained by Oil Red O. Undifferentiated cells were negative (Figure 1(e)). Taken together, these data confirmed the ability of MSCs isolated from the mandibular bone to differentiate towards mesenchymal lineages.

### 3.3. Titanium Surface Characterization

**Figure 2:**
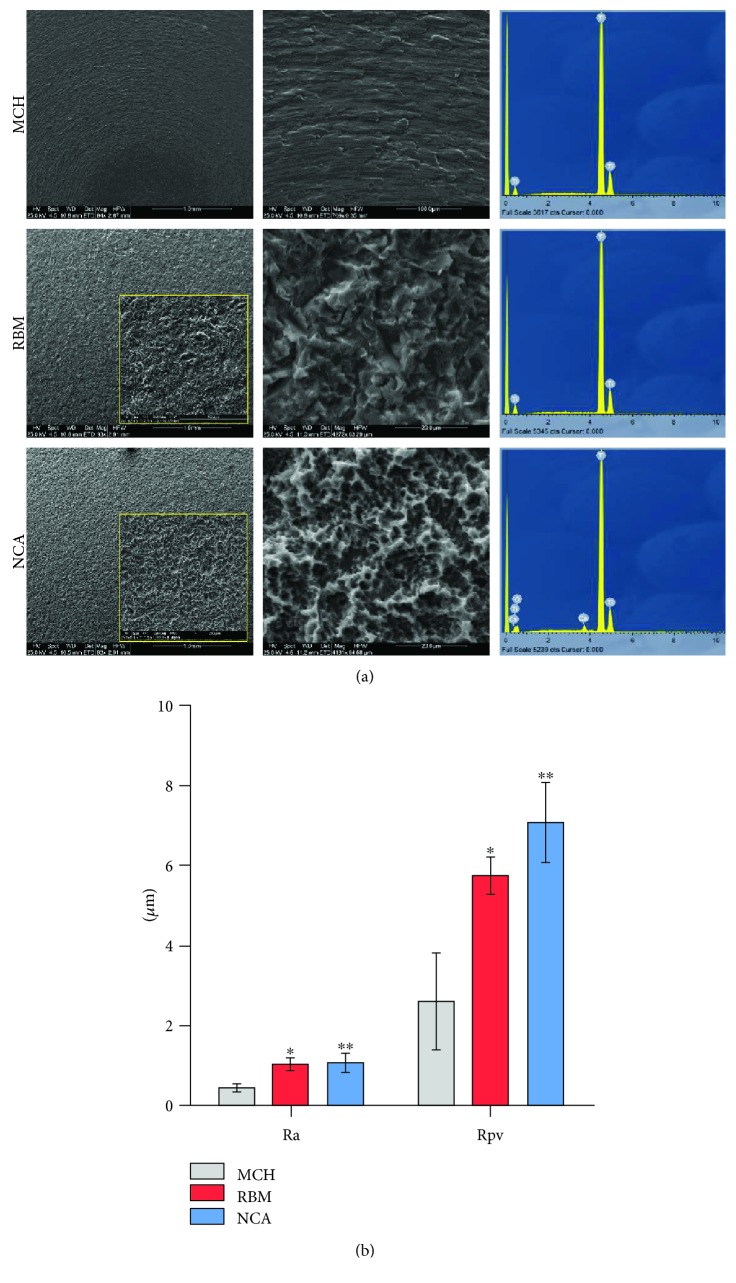
Titanium surface characterization. (a) SEM analysis at different magnifications was carried out on the three types of titanium surfaces (MCH, RBM, and NCA, as indicated) in order to evaluate the surface topography. EDX analysis, reported on the right side of the panel, shows the chemical composition of each analyzed surface. (b) Histograms report the surface roughness expressed as Ra and Rpv values determined by AFM. Values represent mean ± SD of three independent experiments; ^∗∗^ *P* < 0.01, ^∗^ *P* < 0.05 MCH vs. RBM and MCH vs. NCA groups; one-way ANOVA followed by Tukey's comparison test.

SEM analysis carried out on the three types of surfaces is shown in Figure 2. At lower magnifications (95x), MCH surface displayed concentric irregularities while RBM and NCA surfaces were characterized by homogeneous irregularities spread through the whole analyzed areas. At higher magnifications (750x, yellow square), pronounced differences in morphology related to the different surface treatment methods could be better appreciated. 4200x magnification highlighted the differences between the RBM and NCA surfaces. Conversely, from RBM, NCA surface showed a rough nanostructure (Figure 2(a)). EDX analysis, reported on the right side of the panel, showed high peaks for titanium on MCH and RBM surfaces while on NCA surface an additional calcium peak was observed. Histograms in Figure 2(b) reported the surface roughness values determined by AFM. Statistically significant differences between Ra and Rpv parameters in MCH vs. RBM and MCH vs. NCA groups (*P* < 0.05, *P* < 0.01) were detected. No differences were reported in the RBM vs. NCA group in any of the parameters evaluated. Numerical values are reported in Appendix Table 2.

### 3.4. MSC Morphology and Proliferation on Titanium Surface

**Figure 3:**
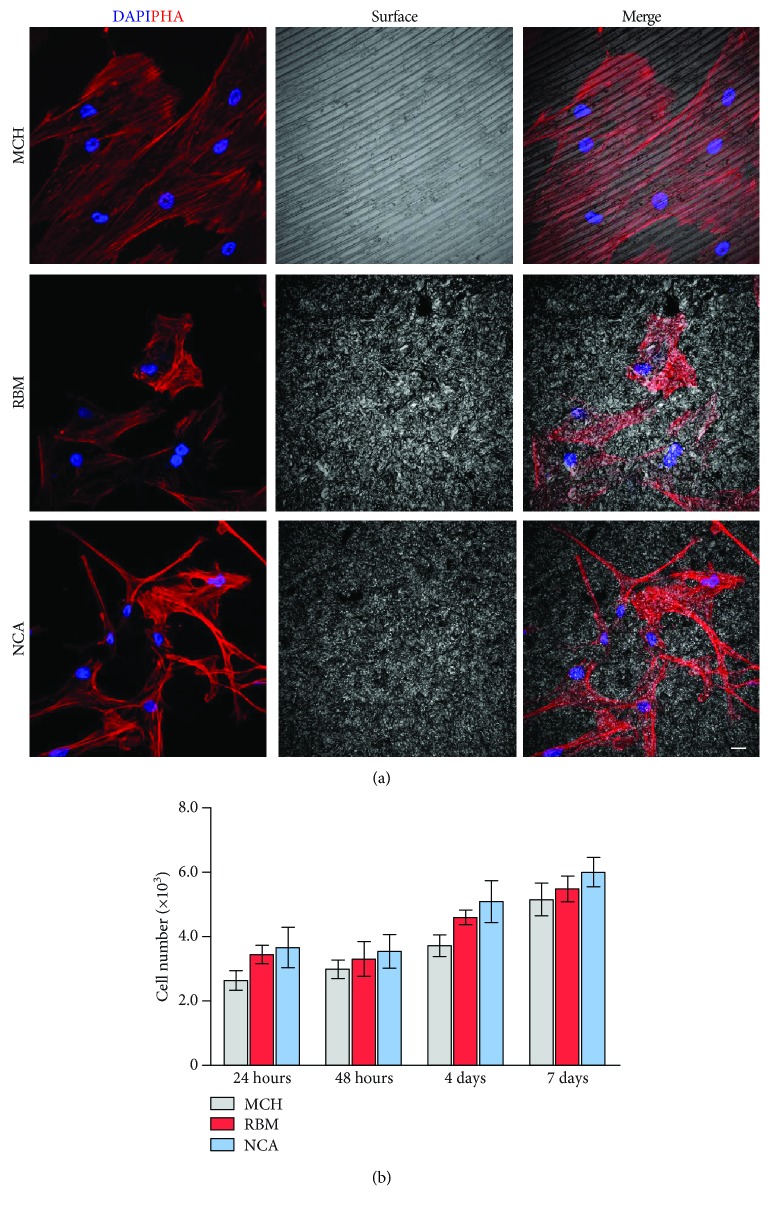
Evaluation of MSC morphology and proliferation on titanium surfaces. (a) MSC morphology was assessed by confocal microscopy on MSCs stained with phalloidin and DAPI at 7 days of culture time on MCH, RBM, and NCA surfaces. (b) Cell proliferation on titanium disks was analyzed by counting cell nuclei following DAPI staining. Histograms show cell numbers after 24 hours, 48 hours, 4 days, and 7 days of culture on the three titanium surfaces. Values represent mean ± SD; No statistically significant difference was detected among the groups at any experimental time; one-way ANOVA followed by the Newman-Keuls post hoc test. Experiments were performed in triplicate. Scale bar: 10 *μ*m.

MSC morphology was assessed by confocal microscopy, as shown in Figure 3(a). Cells were stained with phalloidin and DAPI. After 7 days of culture time, MSCs cultured on the MCH surface displayed an elongated spindle-like morphology with cells being arranged parallel to the surface grooves. In contrast, cells on RBM surface showed a flattened and cuboidal morphology, with cells distributed in a scattered manner through the surface. At the same culture time, MSCs grown on NCA surface showed an irregular shape and a reduction of the average area of a single cell was microscopically appreciable (Figure 3(a)) with cells being spread homogeneously on the entire area. The cell proliferation on titanium disks was analyzed by counting cell nuclei following DAPI staining. The data were expressed as cells per square centimeter and reported in Figure 3(b). As reported in the histogram, there was no statistically significant difference in cell proliferation between the three surfaces at any experimental time. Immunofluorescence analysis performed on MSCs seeded on titanium surface for 24 hours and 4 days was reported in Appendix Figures 1 and 2, respectively.

### 3.5. Osteogenic Induction and FasL Expression

**Figure 4:**
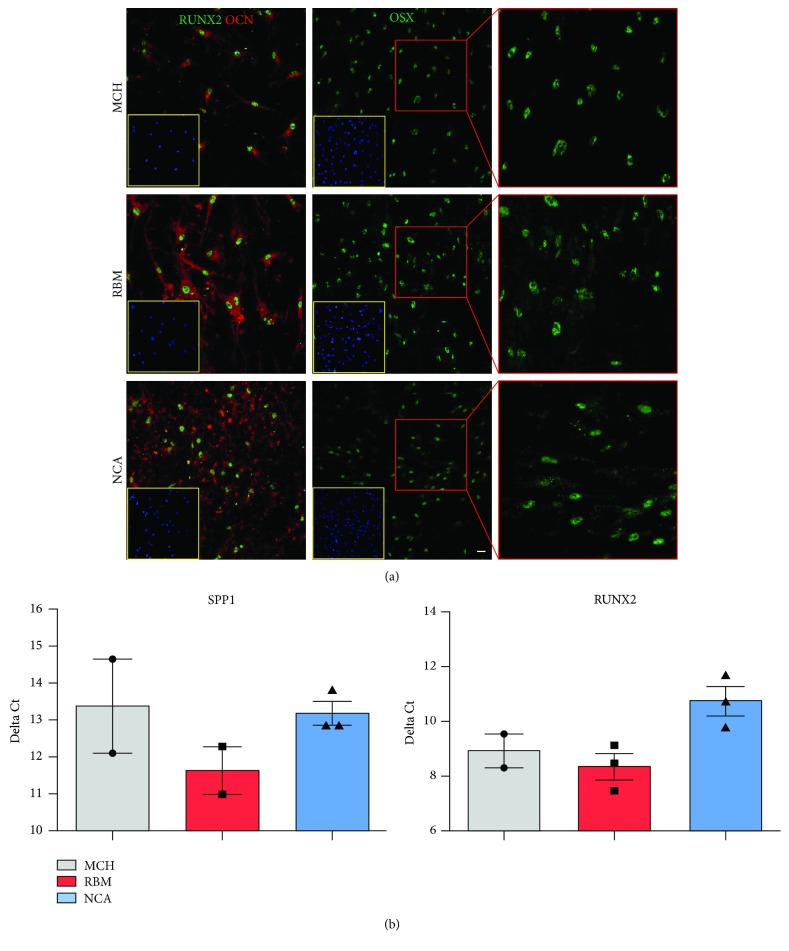
Evaluation of osteogenic induction of MSCs on titanium surfaces. (a) Confocal immunofluorescence analyses of RUNX-2, OCN, and OSX were carried out on MSCs seeded on MCH, RBM, and NCA surfaces, after 3 weeks of osteogenic induction. Nuclei were counterstained with DAPI. Scale bar: 10 *μ*m. (b) Real-time PCR analysis performed on osteogenic differentiated MSCs shows the mRNA expression level relative to GADPH (*∆*Ct) of RUNX-2 and SPP1. Values represent *∆*Ct ± SD; one-way ANOVA followed by the Newman-Keuls post hoc test.

**Figure 5:**
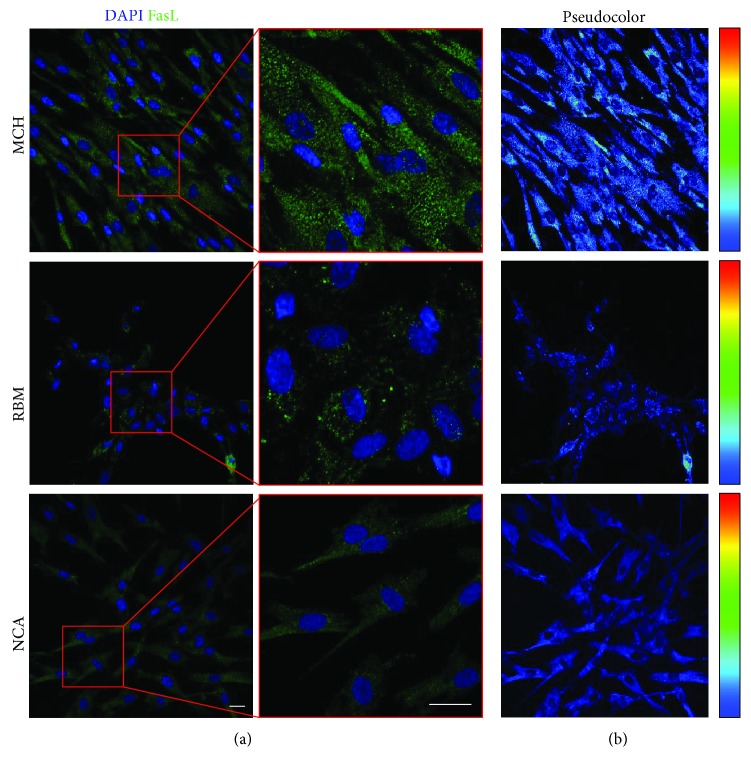
FasL expression in MSCs cultured on titanium surface. Immunofluorescence analysis was carried out on MSCs after 7 days of culture on MCH, RBM, and NCA surfaces. Nuclei were counterstained with DAPI. Scale bar: 10 *μ*m. In (b), pseudocolor analysis of FasL is shown.

After 3 weeks of osteogenic induction, the expression of the osteogenic markers RUNX-2, OCN, and OSX in MSCs seeded on MCH, RBM, and NCA surfaces was evaluated by confocal microscope analysis. After 3 weeks, almost all differentiated MSCs were positive for the three evaluated markers without any significant differences among the three experimental groups (Figure 4(a)). The osteogenic commitment of MSCs was confirmed by real-time PCR analysis with the relative mRNA expressions, reported as *∆*Ct, of RUNX-2 and SPP1 showing no statistically significant differences (Figure 4(b)). As reported in Figure 5, the expression of FasL in MSCs seeded on the three experimental surfaces was evaluated by immunofluorescence analysis. Interestingly, pseudocolor analysis revealed an increase of signal intensity in MSCs after 5 days of culture on MCH. At the same time, an appreciable reduction of the immunofluorescence signals was observed in MSCs cultured on RBM and NCA surfaces.

## 4. Discussion

MSCs can be isolated from almost any tissue in the body, including craniofacial bones [23–25]. These cells exhibit different biological properties when compared to MSCs isolated from axial and appendicular bones. As suggested by Lohberger et al., some bone abnormalities are limited to craniofacial bones assuming that there are significant differences in bone metabolism of orofacial, axial, and appendicular bones [26]. These differences may be dictated by site specificity of embryological progenitor cells and osteogenic properties of resident multipotent MSCs as reported by Akintoye et al. [12]. To this regard, it has been argued that identifying the optimal stem cell source is crucial for a successful cell-based therapy for skeletal healing of different anatomical districts [14].

In light of the appealing potential of MSCs in multiple therapeutic applications, a primary goal is represented by the development of clinical-grade cell preparations that match with the regulatory requirements for cellular therapies under good manufacturing practice-compliant (cGMP) conditions. As a matter of fact, the serum-containing media represent a huge obstacle for MSC-related therapies, due to the risk of contamination by infectious pathogens. Different studies have investigated the use of serum/xenofree culture media to achieve a safe and efficient expansion of mesenchymal stem cells (MSCs) for clinical use [27]. Current strategies also include the replacement of FBS with chemically defined media or with human blood derivatives such as human serum and human platelet lysate [28]. In particular, controversial evidence emerged from studies aimed at identifying suitable alternatives to foetal bovine serum and other animal supplements. Recent findings from Bakopoulou et al. demonstrated that oral BM-MSCs cultured under serum-free conditions showed a downregulation of stemness and MSC markers and the occurrence of cell senescence; moreover, an “osteogenic predisposition” was observed in parallel with a reduced expression of chondrogenic and adipogenic markers [29]. This latter evidence might be favourable for a potential application of MSCs to bone regeneration in dentistry. On the other hand, human platelet lysate (HPL) has been widely investigated proving to be a suitable alternative to foetal bovine serum, mostly for its content in growth factors promoting MSC proliferation [30]. Further, pathogen inactivated HPL has been proposed as a safer candidate to stimulate MSC growth in clinical-scale cultures, since it maintains unaltered proliferation, differentiation, and immunosuppressive properties without affecting the antigenic and functional abilities of MSCs [30, 31]. Different promising experimental settings have been proposed to reach a clinical safety of MSCs ex vivo expansion; nevertheless, there is still a lack of standardized protocols for this goal.

In our study, we have demonstrated that cells isolated from the mandibular bone exhibit the characteristics of cells described as mesenchymal stromal cells in accordance to the criteria proposed by the International Society for Cellular Therapy (ISCT) [9]. The choice of using MSCs isolated from the mandibular bone is driven by the need to find a suitable experimental model to investigate the osseointegration and healing processes occurring during the routine implantology and reconstructive or regenerative surgical procedures. To this regard, the use of craniofacial MSCs cultured with the titanium implants represents a suitable and appropriate experimental approach to better understand the mechanisms of cell interaction with different implantological surfaces. In a recent review, Feller and colleagues described that cellular adhesion, proliferation, and differentiation are influenced by the properties of titanium implants, including their surface microtopography, surface chemistry, or surface energy/wettability [4]. Further studies supported that moderately rough surfaces are able to promote a faster osseointegration when compared to turned implant surfaces [3, 32–34]. By contrast, Esposito et al. found no evidence showing that any particular type of dental implant had superior long-term success. Moreover, findings from Albouy et al. show that rough implants are more susceptible to be affected by peri-implantitis than turned surfaces [35, 36]. In our study, we used three different titanium surfaces in order to better understand whether they can influence the biological properties of MSCs colonizing the dental implants. It is well known that titanium surface properties, including nanoscale morphology and surface chemistry, such as calcium incorporation, may influence MSC behaviour [37, 38]. In fact, beside evident differences in physical and chemical parameters among the three surfaces, we observed different biological properties of MSCs when cultured on MCH, RBM and NCA in terms of cell morphology and distribution on the entire sample area. Evaluation of cellular proliferation did not reveal any statistically significant differences at any experimental time point among the three experimental surfaces. These data are in accordance with the previous study [39] evaluating cell proliferation of MC3T3-E1 on RBM and NCA surfaces at 3, 5, and 7 days. Further, by analyzing cell morphology, we observed that the cellular cytoskeleton organization is influenced by the titanium surface, as shown by the fluorescent staining with phalloidin. Noteworthy, cells cultured on MCH surface were parallel oriented to the grooves, event that was previously described *in vitro* by Anselme et al. and Cipriano et al. and defined as contact guidance [40, 41]. On the contrary, when grown on RBM, surface cells were flat and cuboidal shape, whereas they assumed a highly branched morphology when grown on the NCA surface. These observations are in accordance with findings from Nayab et al. who described similar morphological features *in vitro* in MG-63 cells cultured on calcium ion-implanted titanium scaffolds [42]. According to Nayab et al., cell adhesion, proliferation, and morphology could be affected by Ca ion concentration deposited on titanium surface. Scientific evidences suggest that during MSC osteogenic differentiation, considerable alterations in cell morphology and cytoskeletal organization occur [43]. Although morphological alterations were observed, the expression of osteogenic-related markers revealed by immunofluorescence and PCR analyses was similar among the three experimental groups, suggesting that the three different surfaces did not influence the expression of the osteogenic markers RUNX-2, OSX, SPP1, and OCN at 21 days of induction with the osteogenic medium. These data indicate that surface topography and chemistry do not seem to influence the osteoblastic response of MSCs when kept in favourable differentiating conditions aimed to mimic physiological microenvironment. Even if the different surface features do not seem to play a significant role in MSC osteogenic commitment, interesting results were obtained in the evaluation of FasL expression. In fact, we noticed that cells cultured on MCH expressed high levels of FasL when compared with cells cultured on RBM and NCA. We showed for the first time that the different surface properties are able to influence the immunomodulatory role of MSCs. Immunomodulatory properties of MSCs represent a biological role that needs to be considered when evaluating the cell/biomaterial interaction. The activation of the molecular pathway involved in the modulation of the inflammatory process would help the successfulness of the implant by favouring its osseointegration. In fact, as reported by Ming et al. [18], the upregulation of FasL was demonstrated to strengthen the bone formation by MSC. According to Ming et al., the maintenance of stable or moderately increasing levels of FasL appeared to be primary to keep the balance between proliferation and differentiation of BM-MSCs.

## 5. Conclusions

MSCs isolated from craniofacial bones may represent a suitable model for the study of cellular interaction with titanium surfaces. This model could be applied either to study materials biocompatibility or to evaluate the biological response to biomaterials used in craniofacial regenerative medicine. Differences have been observed between the three surfaces evaluated in terms of morphology and cell distribution. The surface features do not seem to influence the osteogenic marker expression. It is well known that stem cells exhibit many immunomodulatory properties *in vitro* and *in vivo* and are able to modulate the inflammation following the production of soluble factors as well as cell-cell interaction [16]. We have observed for the first time that different implant surfaces are able to influence the expression of FasL in MSCs. FasL is involved in the promotion of osseointegration and bone mass preservation by modulating osteoclasts. Moreover, FasL plays a key role in immunomodulation exerted by MSCs; therefore, it might be of interest to evaluate how this altered expression may influence the host immunological response. It might be concluded that machined surfaces (MCH) allow preserving MSC biological properties similarly to rough surfaces (RBM and NCA). However, MSCs cultured on smooth surfaces showed a higher expression of FasL, suggesting that smooth surfaces may provide favourable conditions for a long-term maintenance of bone mass.

## Data Availability

All the data used to support the findings of this study are included within the article and the Supplementary Materials' file.

## Supplementary Materials

Supplementary Materials This section contains a detailed description of MSC isolation and immunophenotypical characterization besides the multilineage differentiation assays. Two supplementary figures show MSC adhesion to titanium surfaces after 24 hours (Appendix Figure 1) and 4 days (Appendix Figure 2) of culture, and two supplementary tables report data on immunophenotypical characterization of MSCs (Appendix Table 1) and titanium surface roughness parameters (Appendix Table 2).
