# Supplementary material for: Titanium Surface Properties Influence the Biological Activity and FasL Expression of Craniofacial Stromal Cells
Source: Stem Cells Int. 2019 Jan 13;2019:4670560. doi: 10.1155/2019/4670560 (PMC6348805; doi:10.1155/2019/4670560)
Supplement: Supplementary Materials — This section contains a detailed description of MSC isolation and immunophenotypical characterization besides the multilineage differentiation assays. Two supplementary figures show MSC adhesion to titanium surfaces after 24 hours (Appendix Figure 1) and 4 days (Appendix Figure 2) of culture, and two supplementary tables report data on immunophenotypical characterization of MSCs (Appendix Table 1) and titanium surface roughness parameters (Appendix Table 2). [file 4670560.f1.docx]

**Titanium surface properties influence the biological activity and FasL expression of craniofacial stromal cells**

Enrico Conserva ^1,*^, Alessandra Pisciotta ^1,*^, Francesco Borghi ^1,*^, Milena Nasi ^1^, Simone Pecorini ^2^, Laura Bertoni ^1^, Anto de Pol ^1^, Ugo Consolo ^1^, Gianluca Carnevale ^1^

^1^ Department of Surgery, Medicine, Dentistry and Morphological Sciences with interest in Transplant, Oncology and Regenerative Medicine, University of Modena and Reggio Emilia, Modena, Italy; ^2^ Department of Biomedical, Metabolic and Neural Sciences, University of Modena and Reggio Emilia, Modena, Italy.

**Table of contents**

1. **Supplementary materials and methods**

1.1. MSCs isolation and immunophenotypical characterization

1.2. Multilineage differentiation essays
 1.3. Appendix References

**2. Appendix figures and tables**

1. **Supplementary materials and methods**

**1.1. MSCs isolation and immunophenotypical characterization**

MSCs were isolated from mandibular bone as previously described by Carnevale et al. (Carnevale et al. 2016) and reported in Appendix Figure 1. Corticomedullary fresh mandibular bone fragments were obtained from patients (n=3) between 35 and 40 years undergoing routine implant surgery. The study obtained the Ethical Committee’s approval of the province of Modena (Italy) and all patients signed the written informed consent in accordance with the Declaration of Helsinki before the surgery. Bone fragments consisting mainly of bone marrow enclosed in trabecular elements were mechanically disaggregated, placed in culture medium [α-Minimum Essential Medium (α-MEM), 20% fetal bovine serum (FBS) (Euroclone, Milan, Italy), 1% L-glutamine, 1% penicillin and streptomycin (Sigma Aldrich, Saint Louis, MO, USA)] and incubated in standard culture conditions (37°C, 5% CO2, high humidity) upon reaching at least 70% of confluence. During culture, adherent cells were observed in the culture dish after 2 days of incubation. Bone fragments were removed after 2 days.

Following trypsin dissociation, cells were resuspended in culture medium and assayed for their immuno-phenotypical profile by staining with the following fluorochrome-conjugated antibodies (Abs): anti-human-CD73-PE-CY7, -CD90-FITC, -CD105-APC, -CD14-FITC, -CD45-PE, and -HLA-DR-PECY7 (all from BD Biosciences, Franklin Lakes, NJ, USA); and -CD34-ECD (Beckman Coulter, Fullerton, CA, USA). Cells were incubated for 20 min at room temperature and washed with Stain Buffer (BD Biosciences). A minimum of 10,000 cells per sample was acquired and analyzed by using the Attune Acoustic Focusing Flow Cytometer (Attune NxT, Thermo Fisher,Waltham, MA, USA). Data were analyzed by FlowJo 9.5.7 (Treestar, Inc., Ashland, OR, USA) under MacOS 10.

**1.2. Multilineage differentiation assays**

Isolated MSCs were tested for their ability to differentiate toward osteogenic, adipogenic and chondrogenic lineages.

*Osteogenic differentiation.* Osteogenic differentiation was carried out as previously described by Pisciotta et al. and Bianchi et al. (Pisciotta et al. 2012, Bianchi et al. 2017). Cells were seeded at approximately 3x10^3^ cells/cm^2^ on culture dishes and kept in osteogenic medium consisting in α-MEM supplemented with 10% FBS (Euroclone), 100 µM 2P-ascorbic acid, 100nM dexamethasone and 10mM β-glycerophosphate (Sigma Aldrich). After 3 weeks of osteogenic induction extracellular calcium deposition was evaluated by Alizarin Red staining. Undifferentiated hBM-MSCs were used as negative control.

*Adipogenic differentiation.* Cells previously seeded at the cell density of 2 × 10^4^ cells/cm^2^ were incubated for 3 weeks in adipogenic induction medium according to Carnevale et al. (Carnevale et al. 2013) [α-MEM supplemented with 10% FBS (Euroclone), 0.5 mM isobutylmethylxanthine, 1 µM dexamethasone, 10µM insulin, and 200 µM indomethacin (Sigma Aldrich). Lipid-rich vacuoles within the cells were evaluated by Oil red O staining and nuclei were counterstained with haematoxylin. Undifferentiated hBM-MSCs were used as negative control.

*Chondrogenic differentiation.*

Chondrogenic differentiation of the hBM-MSCs was induced using a pellet-culture method. Briefly, the cells were cultured for 3 weeks in polypropylene tubes at a density of 1 × 10^5^ cells/tube in chondrogenic medium consisting in DMEM-HG supplemented with 5% FBS (Euroclone), 100 nM dexamethasone and 10 ng/mL TGF β-1, 10 mM 2P-ascorbic acid (Sigma-Aldrich), 1% v/v sodium pyruvate (Life Technologies, Carlsbad, CA, USA), 50 mg/mL ITS premix (BD Biosciences). To evaluate chondrogenic differentiation, cell pellets were included in paraffin and sections of 5 µm were obtained by microtome. Histological analysis by using Alcyan Blue and Fast Red staining was performed and, to confirm the chondrogenic commitment, immunofluorescence analysis using anti-collagen II antibody (Abcam, Cambridge, UK) was conducted.

**APPENDIX REFERENCES**

Bianchi M, Pisciotta A, Bertoni L, Berni M, Gambardella A, Visani A, Russo A, de Pol A, Carnevale G. 2017. Osteogenic Differentiation of hDPSCs on Biogenic Bone Apatite Thin Films. Stem Cells Int. 3579283.

Carnevale G, Pisciotta A, Riccio M, De Biasi S, Gibellini L, Ferrari A, La Sala GB, Bruzzesi G, Cossarizza A, de Pol A. 2016. Optimized Cryopreservation and Banking of Human Bone-Marrow Fragments and Stem Cells. Biopreservation & Biobanking 14:138–148.

Carnevale G, Riccio M, Pisciotta A, Beretti F, Maraldi T, Zavatti M, Cavallini GM, La Sala GB, Ferrari A, De Pol A. 2013. In vitro differentiation into insulin-producing β-cells of stem cells isolated from human amniotic fluid and dental pulp. Dig Liver Dis. 45:669–676.

Pisciotta A, Riccio M, Carnevale G, Beretti F, Gibellini L, Maraldi T, Cavallini GM, Ferrari A, Bruzzesi G, De Pol A. 2012. [Human serum promotes osteogenic differentiation of human dental pulp stem cells in vitro and in vivo.](https://www.ncbi.nlm.nih.gov/pubmed/23209773) PLoS One. 7(11): e50542.

**APPENDIX FIGURES AND TABLES**

**Appendix Table 1: Quantification of surface markers expression in MSCs**

| Marker | Subject 1  (% of positivity) | Subject 2  (% of positivity) | Subject 3  (% of positivity) |
| --- | --- | --- | --- |
| CD73 | > 99.5 | > 99.5 | > 99.5 |
| CD90 | > 99.5 | > 99.5 | > 99.5 |
| CD105 | > 99.5 | > 99.5 | > 99.5 |
| CD14 | < 0.5 | < 0.5 | < 0.5 |
| CD34 | < 2.0 | < 2.0 | < 2.0 |
| CD45 | < 0.5 | < 0.5 | < 0.5 |
| HLA-DR | < 2.0 | < 2.0 | < 2.0 |

MSCs were obtained from three different healthy subjects. Values represent the percentage of positivity for each marker.

**Appendix table 2**: **Surfaces Roughness parameters**

|  | **MCH** | **RBM** | **NCA** |
| --- | --- | --- | --- |
| **Ra** | 0.436 ±0.102 | 1.047 ±0.150* | 1.086 ±0.246** |
| **Rpv** | 2.607 ±1.200 | 5.750 ±0.454* | 7.085 ±1.014** |

Values represent mean ± SD (µm) of three independent experiment, **P<0.01, *P<0.05 vs MCH; One Way ANOVA followed by Tukey’s comparison test was performed.

**
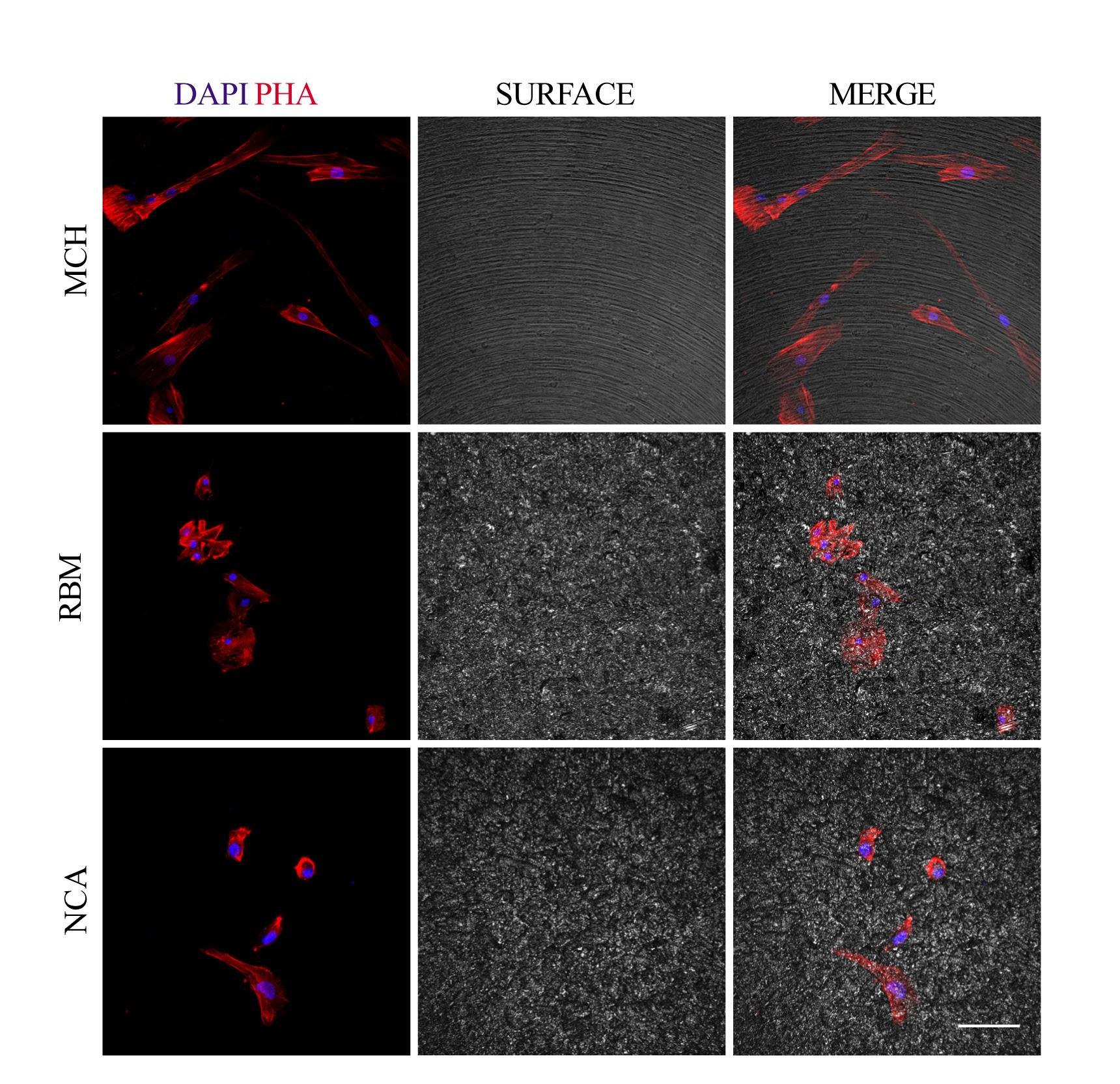
**

**Appendix Figure 1. MSCs adhesion to titanium surfaces after 24 hours of culture.** Confocal microscopy analysis showing MSCs adhesion to MCH, RBM and NCA surfaces as early as after 24 hours of culture. Cells were stained with phalloidin and DAPI. Scale bar. 100 μm.


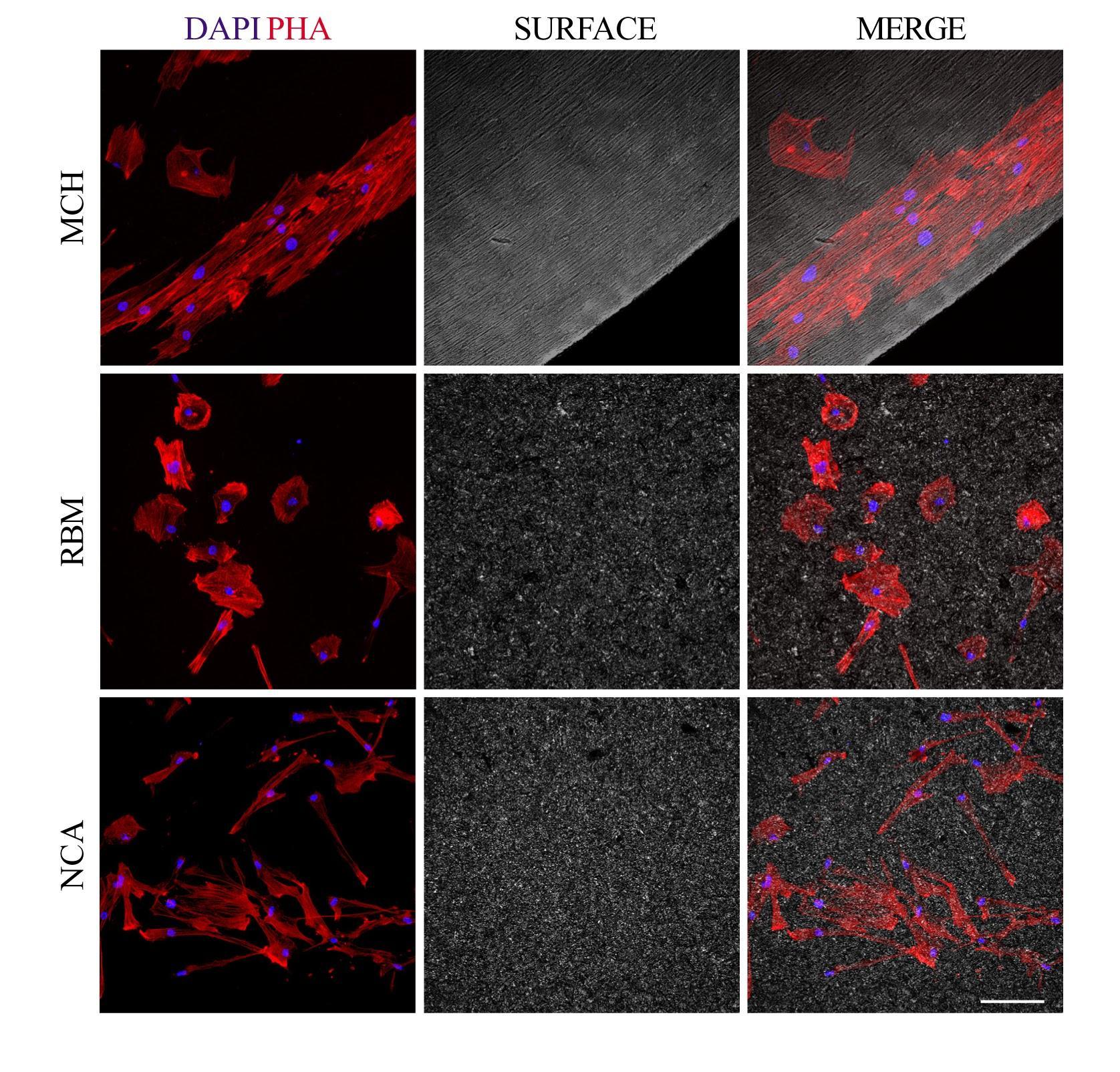


**Appendix Figure 2. Evaluation of MSCs morphology and distribution on titanium surfaces after 4 days of culture.** Confocal microscopy analysis showing MSCs morphology and spread, after 4 days of culture, on MCH, RBM and NCA surfaces. Cells were stained with phalloidin and DAPI. Scale bar. 100 μm.
